# Mutation Spectrum of *EYS* in Spanish Patients with Autosomal Recessive Retinitis Pigmentosa

**DOI:** 10.1002/humu.21334

**Published:** 2010-11

**Authors:** Isabel Barragán, Salud Borrego, Juan Ignacio Pieras, María González-del Pozo, Javier Santoyo, Carmen Ayuso, Montserrat Baiget, José M Millan, Marcela Mena, Mai M Abd El-Aziz, Isabelle Audo, Christina Zeitz, Karin W Littink, Joaquín Dopazo, Shomi S Bhattacharya, Guillermo Antiñolo

**Affiliations:** 1 Unidad de Gestión Clínica de Genética, Reproducción y Medicina Fetal, Instituto de Biomedicina de Sevilla (IBIS), Hospital Universitario Virgen del Rocío/CSIC/ Universidad de SevillaSevilla, Spain; 2 Centro de Investigación Biomédica en Red de Enfermedades Raras (CIBERER)Spain; 3 Departamento de Bioinformática y Genómica, Centro de Investigación Príncipe FelipeValencia, Spain; 4 Departamento de Genética, Instituto de Investigación Sanitaria Fundación Jiménez DíazMadrid, Spain; 5 Servei de Genética, Hospital de la Santa Creu i Sant PauBarcelona, Spain; 6 Unidad de Genética, Hospital Universitari La FeValencia, Spain; 7 UCL-Institute of OphthalmologyBath Street, London, EC1V 9EL. United Kingdom; 8 INSERMU968, Paris, F-75012, France; 9 CNRSUMR_7210. Paris, F-75012, France; 10 UPMC Univ Paris 06, UMR_S 968, Institut de la VisionParis, F-75012, France; 11 Centre Hospitalier National d'Ophtalmologie des Quinze-Vingts, INSERM-DHOS CIC 503Paris, F-75012, France; 12 Department of Molecular Genetics, Institute of OphthalmologyLondon, UK; 13 Department of Human Genetics, Radboud University Nijmegen Medical CentreP.O. Box 9101, 6500 HB Nijmegen, The Netherlands; 14 The Rotterdam Eye HospitalP.O. Box 70030, 3000 LM Rotterdam, The Netherlands; 15 Department of Cellular Therapy and Regenerative Medicine, Andalusian Molecular Biology and Regenerative Medicine Centre (CABIMER)Sevilla, Spain

**Keywords:** *EYS*, Retinitis Pigmentosa, Spanish population, functional domain, recurrent mutation

## Abstract

Retinitis pigmentosa (RP) is a heterogeneous group of inherited retinal dystrophies characterised ultimately by the loss of photoreceptor cells. We have recently identified a new gene (EYS) encoding an ortholog of Drosophila spacemaker (spam) as a commonly mutated gene in autosomal recessive RP. In the present study, we report the identification of 73 sequence variations in EYS, of which 28 are novel. Of these, 42.9% (12/28) are very likely pathogenic, 17.9% (5/28) are possibly pathogenic, whereas 39.3% (11/28) are SNPs. In addition, we have detected 3 pathogenic changes previously reported in other populations. We are also presenting the characterisation of EYS homologues in different species, and a detailed analysis of the EYS domains, with the identification of an interesting novel feature: a putative coiled-coil domain. Majority of the mutations in the arRP patients have been found within the domain structures of EYS. The minimum observed prevalence of distinct EYS mutations in our group of patients is of 15.9% (15/94), confirming a major involvement of EYS in the pathogenesis of arRP in the Spanish population. Along with the detection of three recurrent mutations in Caucasian population, our hypothesis of EYS being the first prevalent gene in arRP has been reinforced in the present study. © 2010 Wiley-Liss, Inc.

## INTRODUCTION

Retinitis pigmentosa (RP) is a heterogeneous group of inherited retinal dystrophies featured by the loss of photoreceptor cells and clinically characterized by pigmentory deposits at mid periphery of the retina that are visible on fundus examination. Patients present with night blindness as the initial symptom, which is followed by the constriction of the visual field and progressive loss of visual acuity, leading to complete blindness after several decades [Hamel, 2006]. Prevalence of nonsyndromic RP is approximately 1 in 4000. The condition may segregate as an autosomal dominant, autosomal recessive, or an X-linked recessive trait [Humphries et al., 1990]. All genes identified to date are believed to account for roughly 50% of all retinal dystrophy cases [Pomares et al., 2007]. The autosomal recessive form of RP is the commonest worldwide, accounting for approximately 39% of cases in Spain [Ayuso et al., 1995]. To date, 29 loci have been reported being responsible for arRP, of which 25 genes have been identified (http://www.sph.uth.tm.edu/Retnet/). However, all together the reported loci are responsible for only ∼35-45% of the recessive RP cases, although none of them independently account for a substantial proportion of arRP (more than 10%) [Daiger et al., 2007; Hartong et al., 2006]. In contrast, the *RP25* locus, identified by our group in 1998 [Ruiz et al., 1998], was estimated to be linked to 27.7% of Spanish arRP families [Barragán et al., 2008]. Recently, we have identified a new gene encoding an ortholog of *Drosophila* spacemaker (spam) corresponding to *RP25* as a commonly mutated gene in arRP. The identification of six independent mutations, together with the presence of linked families from different ancestral origins, supports *EYS (Eyes Shut Homologue*, (MIM# 612424) as one of the first major genes reported for arRP [Abd El-Aziz et al., 2008]. Spanning over 2 Mb within the *RP25* locus (6p12.1-6q15), *EYS* is the largest gene identified to be expressed in the human eye so far, and the fifth largest gene overall in the human genome. The longest isoform of *EYS* encodes a protein of 3165 amino acids whose function remains to be elucidated. Considering the evolutionary data and the known function of the only characterised homologue, EYS is likely to have a role in the modelling of retinal architecture [Zelhof et al., 2006]. The identification of the gene for *RP25* reveals what might be the genetic basis for a significant proportion of arRP cases and thus paves the way for genetic counselling, prenatal detection, and treatment. However, further characterisation of the novel EYS protein as well as an extended mutation spectrum of *EYS*-related arRP would be valuable to undertake.

In the present study based on 94 families, we report the identification of 73 sequence variations in *EYS*, of which 28 are novel. Of these novel changes, 42.9% (12/28) are very likely pathogenic, 17.9% (5/28) are possibly pathogenic, whereas 39.3% (11/28) are SNPs. In addition, we have detected 3 pathogenic changes previously reported in other populations. The estimated prevalence of distinct *EYS* mutations in our group of patients is of 15.9%, confirming the significant involvement of *EYS* in the pathogenesis of the arRP in the Spanish population. Besides, we present a detailed bioinformatic characterisation of EYS and its homologues, which would aid in the determination of the pathogenic nature of newly identified variations in *EYS*.

## MATERIALS AND METHODS

### Subjects and Clinical Data

Our current cohort of study comprises 94 unrelated Spanish families affected by arRP, all derived from the Ophthalmology Service of different Hospitals throughout Spain. The participating families conform to the phenotypic and inheritance patterns of arRP. A group of matching control individuals was also recruited. Informed consent was obtained from all participants in the study, in accordance with the tenets of the Declaration of Helsinki (Edinburgh, 2000). Clinical diagnosis was based on visual acuity, fundus photography, computerized testing of central and peripheral visual fields and electroretinography (ERG) findings. Clinical features of RP include initial hemeralopy, restriction of visual field, gradual increased bone spicule pigmentation and decrease of visual acuity, attenuation of retinal vessels, and waxy disc pallor.

### Bioinformatic characterisation of EYS

Firstly, ExPASy ProtParam tool was employed to determine the physical and chemical parameters of EYS. Secondly, InterProScan program was used to search for known domains and functional sites within EYS. For a further characterisation of EYS, we used Coils and Secpred to analyse the secondary structure. SignalP 3.0 was utilised to predict the presence and localisation of signal peptide cleavage sites. The characterisation of EYS homologues was performed in different steps. Firstly, Blast analyses of the human *EYS* cDNA and encoded protein were run to detect annotated homologous proteins. However, only human and *Drosophila* were found to be completely annotated in the databases. Therefore, we employed the BLAT tool at the UCSC Genome Bioinformatics Site to identify and map sequences with high identity to the target sequences. Human and *Drosophila* EYS protein were used as the basis of this search. An *in-silico* splicing site characterisation of the positive genomic region ensued to build the homologous cDNA which was then translated into protein. Also, we identified those sequencing gaps within the genomic region of *EYS* homologues in different species. Comparison of protein homologues were performed using bl2seq (NCBI) and EMBOSS Pairwise Alignment Algorithms: Needle and Water (EBI) alignments. The fully characterised proteins were aligned using MUSCLE (**MU**ltiple **S**equence **C**omparison by **L**og-**E**xpectation) program at EBI.

### PCR-based direct genomic sequencing of EYS

Peripheral blood samples were collected from all subjects for genomic DNA purification using an automated DNA extractor (MagNA Pure LC Instrument, Roche Diagnostics, Switzerland). Forty-eight pairs of primers were designed using the Primer 3 Output program (http://frodo.wi.mit.edu/primer3/) in order to screen the forty coding exons, the three non coding exons, the intronic flanking sequences and the regulatory factor binding sites of *EYS* (GenBank Reference Sequence and Version FJ416331; GI: 212675237; Transcript Reference Sequence: NM_00 1142800.1). PCR conditions and primer sequences employed are available upon request. The amplified products were subsequently purified using an enzymatic procedure, according to manufacture's recommendations (EXOSAP-IT®, USB Corporation) and sequenced with a ready reaction kit (BigDye Terminator Cycle FS Ready Reaction Kit; PE-Applied Biosystems, Foster City, CA). The fragments obtained were purified using fine columns (Sephadex G-501, Sigma-Aldrich Co.) and resolved on an automated sequencer (3730 DNA Analyzer, Applied Biosystems, USA). Finally, the data was analysed using Lasergene DNASTAR® software (DNASTAR, Inc). Nucleotide numbering reflects cDNA numbering with +1 corresponding to the A of the ATG translation initiation codon in the reference sequence, according to journal guidelines (http://www.hgvs.org/mutnomen). The initiation codon is codon 1. In order to evaluate the pathogenicity of the novel variants, we employed various softwares which analyse the potential role of a given variant on the function or structure of the encoded protein based on conservation and homology, physical properties of the amino acids, prediction of the protein disorder, or binding to transcription factor binding sites (TFBS) (Conseq: http://conseq.tau.ac.il/; PolyPhen (prediction of functional effect of human nsSNPs): http://coot.embl.de/PolyPhen/; SIFT (Sorting Intolerant From Tolerant): http://blocks.fhcrc.org/sift/SIFT.html; Disopred: http://bioinf.cs.ucl.ac.uk/disopred/disopred.html [Ramensky et al., 2002]. Besides, the tool DiANNA was employed for disulfide connectivity prediction when the variation affected a Cys residue, andNetPhos2.1 (NetPhos 2.0 Server: http://www.cbs.dtu.dk/services/NetPhos/) together with Diphos (Disorder-Enhanced Phosphorylation Sites Predictor: http://core.ist.temple.edu/pred/pred.html), to predict the alteration of phosphorylation [Blom et al., 1999]. In addition, intronic variants were evaluated for affecting any regulatory process at the transcriptional or splicing levels (TESS Transcription Element Search System: http://www.cbil.upenn.edu/cgi-bin/tess/tess; http://www.fruitfly.org/seqtools/splice.html; Splice SignalAnalysis: http://www.ebi.ac.uk/asd-srv/wb.cgi; Alternative Splicing DataBase: http://hazelt0n.lbl.g0v/∼ teplitski/alt/; http:/www.fruitfly.org/cgi-bin/seqtools/splice.pl; Splicing Element Annotation: http://genes.mit.edu/acescan2/index.html; ESEfinder: http://rulai.cshl.edu/tools/ESE2/; Rescue-ESE: http://genes.mit.edu/burgelab/rescue-ese/; ACESCAN2 Web Server: http://genes.mit.edu/acescan2/index.html; NetGene2 Server (http://www.cbs.dtu.dk/services/NetGene2/; http://www.ensembl.org/Homosapiens/generegulationview; http://www.cisred.org/content/software; http://regrna.mbc.nctu.edu.tw/html/about.html. [Yeo et al., 2004; Matlin et al., 2005; Wang & Marin 2006; Fairbrother et al., 2002; Brunak et al., 1991].

### GeneChip 6.0 data analysis (see Affymetrix Genotyping Console 2.1 user manual for details)

When sample requirements were fulfilled, patients underwent Copy Number Variations (CNVs) analysis with Genechip 6.0 Affymetrix array. The CEL intensity files were loaded into Genotyping Console v2.1 (Affymetrix Inc.) for analysis. All samples passed the initial contrast QC metric (>0.4) that measures the ability of the intensity files to resolve SNPs into three genotyping clusters. Copy number data were generated by comparing intensities for both SNP and copy number probes *in silico* to the HapMap control provided by Affymetrix. The resulting log2 ratios were then analysed using a Hidden Markov Model (HMM) to generate copy number calls for each probe. The quality of the log2 data was assessed by the degree of variation, determined by the MAPD metric. MAPD is defined as the Median of the Absolute value of all Pairwise Differences between log2 ratios for a given chip. High MAPD >0.4 (using the HapMap control) is considered to be the cut-off at which copy numbers can no longer be accurately called. None of the samples included in this study had a MAPD >0.4. Using the copy number calls provided by Genotyping Console v2.1 as a guide, a more detailed analysis was performed by interrogation of the plots of log2 ratios paying particular attention to CNV regions called by the HMM. A minimum log2 ratio cut-off of +/-0.3 was used for autosomal CNVs. This excluded any false positive calls made by the HMM algorithm. Identified CNVs in the region of *EYS* were checked against the Database of Genomic Variants (DGV) in Genotyping Console v2.1. CNVs were excluded from further analysis if they matched a known CNV exactly, or if occurred in a region that did not include any of the coding exons of *EYS* [McCarroll et al., 2006; de Smith et al., 2007; Sharp et al., 2005]. The allelic difference and loss of heterozygosity plots generated from the difference in allele intensity for each SNP were analysed to investigate parental consanguinity and as an aid to the interpretation of potential mosaicism. Genotyping was performed using the Birdseed v.2 algorithm. All samples had call rates >97.5%.

## RESULTS AND DISCUSSION

Molecular diagnosis of RP is a challenging task given the important genetic heterogeneity of these groups of diseases. For most genes, many different mutations with similar consequences are known, yet other mutations in the same gene may cause different diseases. Particularly, for recessive RP, which is the most prevalent form of the disease, estimated to comprise from 50 to 60% of all RP cases, 29 loci have been described as pathogenic. In aggregate, the known mutations in arRP genes cause about ∼35-45% of all cases of this form of the disease [Daiger et al., 2007; Hartong et al., 2006]. Some authors suggest that 50-60% of all arRP associated causal loci have already been identified. However, mutations in individual genes do not account for a significant proportion of arRP cases.

*EYS* is the largest gene identified to be expressed in the human eye so far, and appears to be a frequent cause of arRP. Thus, *EYS* encoded protein EYS emerges as a relevant player in arRP pathogenesis. In previous studies, a total of 8 mutations had been identified [Abd El-Aziz et al., 2008; Collin et al., 2008] by a combination of different screening methods such as direct genomic sequencing, MLPA or CGH arrays. The domain structure of EYS has been predicted from the characterised sequence of *EYS* [Abd El-Aziz et al., 2008; Collin et al., 2008] as having several EGF-like and Laminin G domains.

Here we report on the molecular screening of *EYS* in a Spanish cohort of patients with arRP. Besides, we are presenting the characterisation of EYS homologues in different species, and a detailed analysis of the EYS domains, with the identification of an interesting novel feature.

### Protein domains structure

**Figure 1:**
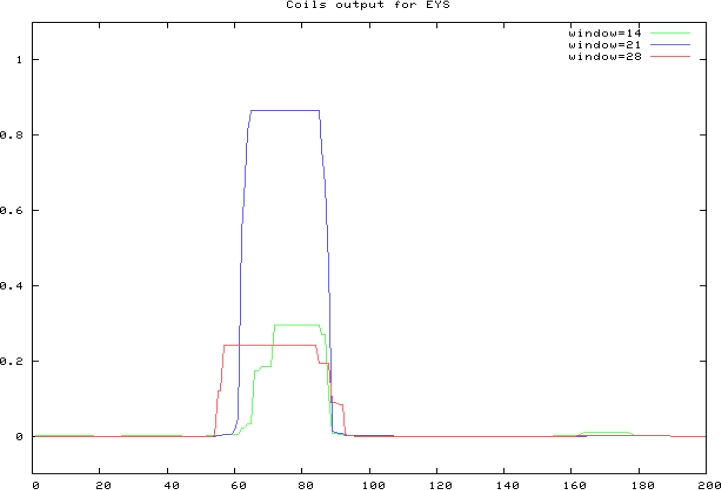
Coiled coil domain prediction in human EYS protein.

**Figure 2:**
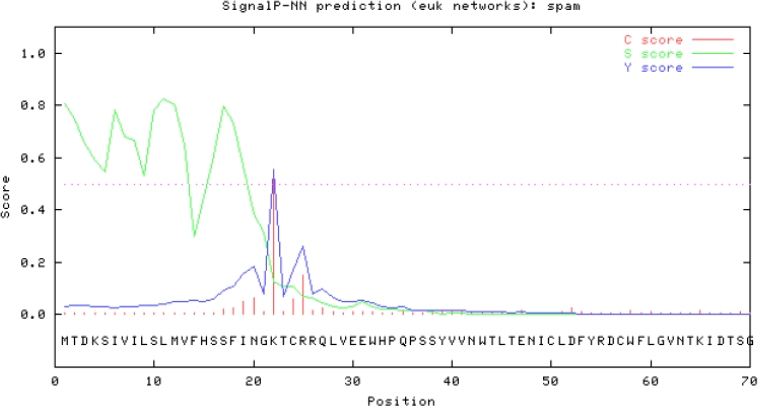
Signal peptide and cleavage site consensus sequence prediction in human EYS protein.

We present here the identification of a putative coiled-coil structure, which is an interesting novel feature, in the central portion of the protein coincident with a region of Alpha helix overrepresentation (Fig. 1, Supp. Figure S1). The insight into the functional repercussion of both the already known signal peptide domain and the novel coiled-coil domain reported here supports a structural role for this new protein, which would be secreted and polymerize into a scaffolding that would contribute to the human retinal architecture. This is consistent with the function of *Eys* in *Drosophila*, where it is secreted by photoreceptor cells [Husain et al., 2006] and is essential for the formation of the matrix-filled interrhabdomeral space. The signal peptide and its cleavage site consensus sequence located in the N-terminal region of EYS (Fig. 1, Fig. 2) may confer a secretory nature to the protein or result in an intracellular or cytoplasmatic location of the mature protein [Jarjanazi et al., 2008]. Remarkably, we have identified the consensus sequence for this feature in *EYS* homologues in a number of species such as orangutan, dog, horse, marmoset, monkey and chimpanzee (data available on request). Accordingly, *Drosophila* Spacemaker and other proteins which share several of EYS domains have been found to be secreted and to have a structural function, such as SCUBE [Yang et al., 2002] or CMG-2 [Bell et al., 2001]. The members of SCUBE gene family contain both a signal peptide domain and multiple EGF-like repeats. Interestingly, both EYS and SCUBE1 share homology with the same protein families, such as members of the fibrillin and Notch families among others. SCUBE1 and 2 are known to form oligomers and manifest a stable association with the cell surface in vascular endothelial cells [Yang et al., 2002]. CMG-2, containing a potential signal peptide, targets to the endoplasmic reticulum and shows affinity for the basement membrane matrix proteins, collagen type IV and laminin. Similarly, CMG-1, which encodes a protein with coiled-coil domains, was observed to target to an intracellular vesicular compartment and may play as well a structural role since it has been postulated that this gene may be implicated in the regulation of capillary formation in an *in-vitro* model of endothelial cell morphogenesis [Bell et al., 2001].

Furthermore, the fact that we have identified a putative coiled-coil domain within EYS reinforces the idea of EYS being a key player in the organisation of human retina. Coiled coils are important structural motifs involved in a variety of important interactions [Mason & Arndt, 2004]. Dystrophin, which resembles *EYS* in several aspects such as presenting a point mutation/deletion pattern of mutations in human disease and belonging to the group of five longest genes in the human genome, is known to play structural roles among others. Dystrophin also contains coiled-coil domains, which are responsible for the assembly of heterodimers of the so called Dystrophin glycoprotein complex [Sadoulet et al., 1997; Böhm et al., 2008]. In this regard, it is important to mention that *Drosophila* Spacemaker interacts with Prominin and the cell adhesion molecule Chaoptin to choreograph the partitioning of rhabdomeres into an open system, critically affecting retinal morphogenesis [Zelhof et al., 2006].

### Annotation of new homologues

With the aim of evaluating evolutionary conservation, we have performed the bioinformatic characterisation of *EYS* homologues in several species. Apart from *Drosophila Eyes Shut*, which was the first *Eys* gene to be annotated, and human *EYS*, only fragmented information was available of other *EYS* homologues. Here we report in detail the structures of Zebrafish, Chicken, Platypus, Opossum, Horse, Dog, Marmoset and Orangutan homologues and pairwise comparisons with human *EYS/EYS* (Table 1, Supp. Figure S1). However, EYS seems to be absent in some species such as those insects with a close rhabdom system or in mammals in mouse, rat and guinea pig, which represent two of the three major rodent clades [Abd El-Aziz et al., 2008].

**Table 1:** *EYS* homologues characterisation

Specie	Chromosomal interval (bp)	Genomic Length (bp)	Genomic Identity%	Protein Identity%	Protein Similarity%
**Drosophila**	chr2L:2323799-2357874	34,076	NA	23.9	37.9
**Zebrafish**	chr13:37105545-37227793	122,249	NA	44.0	60.3
**Chicken**	chr3:87827859-88369092	541,234	71.9	33.1	40.3
**Platypus**	chr1:33486985-34313969	1,756,349	71.7	31.8	36.6
**Opossum**	chr2:312522958-314990462	2,467,505	71.9	35.1	40.5
**Horse**	chr20:56733300-58137441	4,247,273	80.5	61.4	67.1
**Dog**	chr12:30185993-31709408	1,701,147	81.9	62.8	68.4
**Marmoset**	Several contigs*	973,997	91.4	88.8	92.2
**Orangutan**	chr6:63945057-65776065	1,831,009	97.3	96.8	97.5
**Human**	chr6:64488454-66262024	1,773,571	100.0	100.0	100.0

* Available on request

NA=Not Available

It is noteworthy that the previously reported and newly identified functional domains described here are conserved throughout evolution. Consistently, as shown in Table 1, the more distant a species is from human, the lower the percentages of identity and similarity. Concerning the signal peptide in *Drosophila*, some studies report that spacemaker secretion would be upon interaction of *Eys* with a receptor, which could promote its spreading from the stalk to the rhabdomere to fill the interrhabdomeral space (IRS) [Husain et al., 2006]. Thus, there would be no need of a signal peptide for secretion from the photoreceptor cells.

### Pathogenic nature of the identified changes

**Figure 3:**
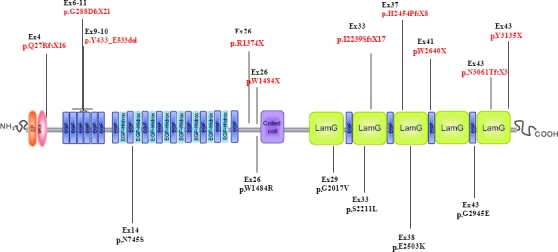
*EYS* mutation distribution along the domain structure of *EYS*/EYS. 5' UTR and splice site variations are not included in this depiction.

**Figure 4:**
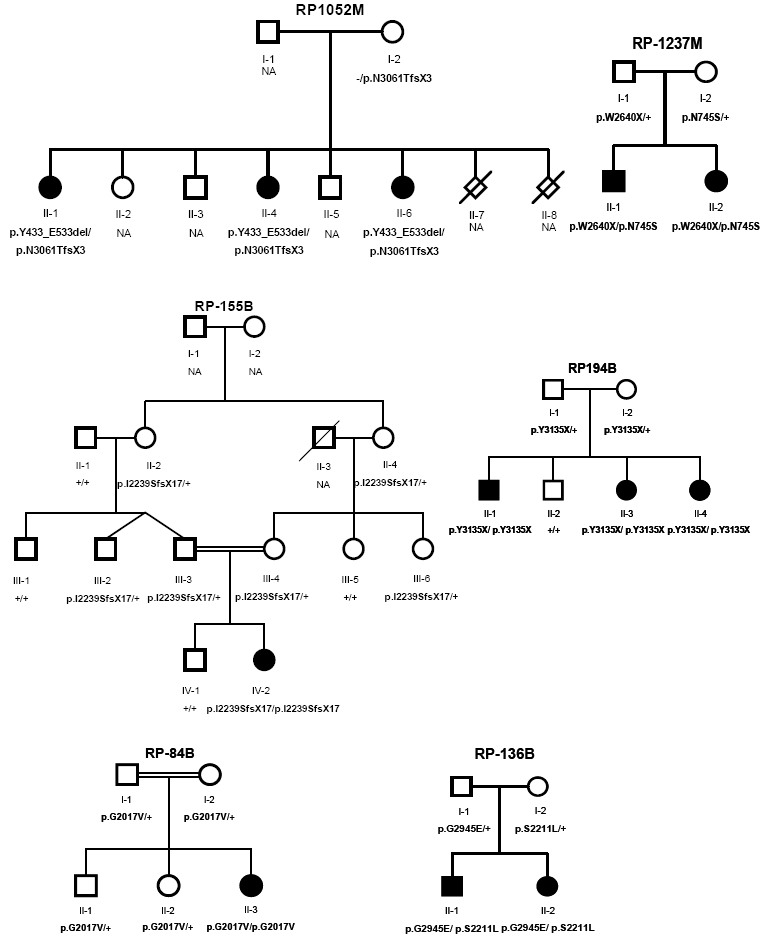

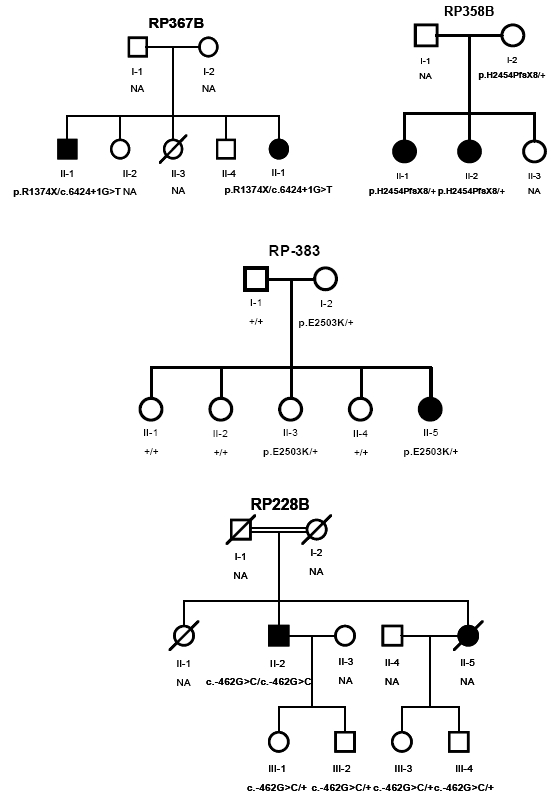
Family segregation of variations identified in the studied arRP families. Below the individuals, genotypes are presented for each change detected to segregate with the RP. For example, p.G2017V/p.G2017V represents homozygous mutants; p.G2017V/+ indicates heterozygous carriers, +/+ indicates individuals carrying two wild-type alleles, whereas p.R1374X/c.6424+1G>T represents individuals presenting both mutations as compound heterozygous. NA means non available DNA sample.

In this study, 12 novel very likely pathogenic changes have been identified in 10 families. Of these 10 families, 5 present mutations in both alleles, whereas the remaining 5 have mutations in just one allele. The clearly pathogenic variants consisted of 6 truncating mutations, 1 in frame deletion of 300 nucleotides leading to a protein truncation of 100 aminoacids, 1 splice site mutation and 4 missense changes. Out of the 28 novel variations, we have also identified 5 possible pathogenic changes in 5 separate families. In addition, we have detected 3 pathogenic variations previously published in ours and other populations [Abd El-Aziz et al., 2008; Collin et al., 2008] (Table 2, Table 3, Figs. 3 and 4). As mentioned in the Methods section, the sequence variants were designated in accordance with the Human Genome Variation Society recommendations (http://www.hgvs.org/mutnomen/). All the patients with mutations had received a defined clinical diagnosis of RP with a recessive mode of inheritance and were Spanish. The variations were regarded as pathogenic changes as long as they met the criteria of pathogenicity, i.e absence in 200 control individuals and the segregation with the disease phenotype within the family (Fig. 4). Particularly, missense mutations were considered pathogenic according to their effect on functional EYS domains that they target, their evolutionary conservation and/or to the fact that they are found together with a second variant, especially if this is truncating.

**Table 2:** Mutation spectrum of *EYS* gene in Spanish families

Family ID	Nucleotide change	Predicted effect	Domains	Location in gene	Type of change	Reference of the variation
Families with novel very likely pathogenic changes and both alleles affected						
RP1052M	c.[1300-17039_1599+22208del]+[9178_9181delATAA]	p.Y433_E533del	EGF-like	Exons 9 and 10	Heterozygous	This study
		p.N3061TfsX3	LamininG	Exon 43	Heterozygous	This study

RP1237M	c.[2234A>G] + [7919G>A]	p.N745S	EGF	Exon 14	Heterozygous	This study
		p.W2640X	EGF	Exon 41	Heterozygous	This study and Abd El-Aziz et al., 2008

RP84B	c.6050G>T	p.G2017V	LamininG	Exon 29	Homozygous	This study

RP136B	c.[6632C>T] + [8834G>A]	p.S2211L + p.G2945E	LamininG	Exon 33	Heterozygous	This study
			EGF	Exon 43	Heterozygous	This study

RP367B	c.[4120C>T] + [6424+1G>T]	p.R1374X	Close to EGF	Exon 26	Heterozygous	This study
		Splice mutation		Intron 31	Heterozygous	This study

Family ID	Nucleotide change	Predicted effect	Domains	Location in gene	Type of change	Reference of the variation
Families with single novel very likely pathogenic changess						
RP358B	c.7361delA	p.H2454PfsX8	LamininG	Exon 37	Heterozygous	This study

RP60	c.78_79dupGC	p.Q27RfsX16	Signal peptide cleavage site	Exon 4	Heterozygous	This study

RP180M	c.862-10671_1766+100 20del	p.G288DfsX21	EGF	Exons 6-11	Heterozygous	This study

RP33	c.4451G>A	p.W1484X	Close to coiled-coil	Exon 26	Heterozygous	This study

RP81	c.2234A>G	p.N745S	EGF	Exon 14	Heterozygous	This study

Families with novel possible pathogenic changes						
VRP8	c.4450T>C	p.W1484R	Close to coiled-coil	Exon 26	Heterozygous	This study

RP383	c.7507G>A	p.E2503K	LamininG	Exon 38	Heterozygous	This study

RP228B	c.-462G>C	-	-	Exon 1 5′UTR	Homozygous	This study

RP107B	c.-204G>A	-	-	Exon 3 5′UTR	Heterozygous	This study

RP509M	c.-349G>T	-	-	Exon 2 5′UTR	Heterozygous	This study

Families with very likely pathogenic changes reported in other populations						
RP155B	c.6714delT	p.I2239SfsX17	LamininG	Exon 33	Homozygous	This study Collin et al., 2008.

RP194B	c.9405T>A	p.Y3135X	LamininG	Exon 43	Homozygous	This study Collin et al., 2008.

GenBank Reference Sequence and Version FJ416331; GI: 212675237; Transcript Reference Sequence: NM_001142800.1 Nucleotide numbering reflects cDNA numbering with +1 corresponding to the A of the ATG translation initiation codon in the reference sequence, according to journal guidelines (http://www.hgvs.org/mutnomen). The initiation codon is codon 1.

**Table 3:** Novel variations identified by direct genomic sequencing of *EYS*

Gene_exon	Nucleotide Change	Predicted effect	Patients Frequency- Controls frequency	
*EYS*_1	c.-462G>C	-	2/188	0/400

*EYS*_2	c.-349G>T	-	1/188	0/400

*EYS*_3	c.-204G>A	-	2/188	0/400

*EYS_4*	c.748+209delA	-	1/188	-
	c.78_79dupGC	p.Q27RfsX16	1/188	0/400

*EYS*_6-11	c.862-10671_1766+10020del	p.G288DfsX21	2/12	0/400

*EYS*_9-10	c.1300-17039_1599+22208del	p.Y433_E533del	2/12	0/400

*EYS*_11	c.1766+61A>G	-	7/188	-

*EYS*_13	c.2024-14_-13insT	-	13/188	-

*EYS*_14	c.2234A>G	p.N745S	2/188	0/400

*EYS*_17+18	c.2733T>C	p.N911N	1/188	-

*EYS*_24	c.3684+61T>A	-	2/188	-

*EYS*_25	c.3877+18_22delAGATA	-	10/188	68/400

*EYS*_26	c.4450T>C	p.W1484R	1/188	0/400
	c.4451G>A	p.W1484X	1/188	0/400
	c.4120C>T	p.R1374X	1/188	0/400

*EYS*_29	c.6050G>T	p.G2017V	1/188	0/400
	c.5959A>C	T1987P	1/188	2/400

*EYS*_30	c.6119T>A	p.V2040D	1/188	2/400

*EYS*_31	c.6424+1G>T	-	1/188	0/400

*EYS*_33	c.6632C>T	p.S2211L	1/188	0/400

*EYS*_37	c.7361delA	p.H2454PfsX8	1/188	0/400

*EYS*_38	c.7507G>A	p.E2503K	1/188	0/400
	c.7578+18C>T	-	2/188	6/400

*EYS*_39	c.7666A>T	p.S2556C	2/188	20/400
	c.7723+64T>A	-	3/188	34/400

*EYS*_43	c.9178_9181delATAA	p.N3061TfsX3	1/188	0/400
	c.8834G>A	p.G2945E	1/188	0/400

GenBank Reference Sequence and Version FJ416331; GI: 212675237; Transcript Reference Sequence: NM_001142800.1 Nucleotide numbering reflects cDNA numbering with +1 corresponding to the A of the ATG translation initiation codon in the reference sequence, according to journal guidelines (http://www.hgvs.org/mutnomen). The initiation codon is codon 1.

### Families with novel very likely pathogenic changes and both alleles affected

As previously mentioned, 5 out of the 10 families bearing novel very likely pathogenic changes have both alleles affected. In 4 of them, they occurred as compound heterozygotes and hence it is sufficient to explain the recessive phenotype in their corresponding patients (Table 2, Fig. 4). In the case of family RP1052M, 1 frameshift deletion of 4 nucleotides involving the loss of the last residues of the Laminin G domain in the C-terminal region of EYS together with an in frame deletion of 300 nucleotides leading to a protein truncation of 100 amino acid residues that disrupts one EGF-like domain were identified in the same patient. In family RP1237M, the truncating p.W2640X change and the missense p.N745S variation, also occurring in heterozygosity in RP81 family, are showing a recessive segregation through generations I and II. The same applies to p.S2211L and p.G2945E double heterozygotes in family RP136B (Fig. 4). These protein substitutions p. S2211L and p.G2945E are each transmitted by one of the progenitors. The altered residues are part of Laminin G and EGF domains, respectively. Turning to evolutionary conservation, both Serine and Glycine are present in these positions in *EYS* homologues characterised in this study. The new residue at amino acid position 2211 is of different polarity than Serine, and the substitution of Glycine to Glutamate in position 2945 introduces an acidic polarity in a previously hydrophobic position. Whereas the latter is not tolerated according to computational predictions, the former implies the loss of one phosphorylation site (NetPhos2.1). Finally, all affected members of family RP367B were compound heterozygous for a splice site and a nonsense mutation (c.6424+1G>T, p.R1374X) (Fig. 4). The c.6424+1G>T variation is predicted to lead to an abolishment of the donor splice site located at this position. It is known that splice sites mutations may disrupt protein function by diverse mechanisms such as exon skipping or the use of cryptic acceptor sites, presenting even multiple splice outcomes for a mutation in a given splice site [Takahara et al., 2002].

The fifth family of this group, RP84B, presents a homozygous coding variation, p.G2017V, which alters a residue that lies within a Laminin G domain of the protein and it is predicted not to be tolerated by SIFT. Family segregation of this variation shows a transmission pattern compatible with the recessive trait of the disease (Fig. 4).

### Families with single novel very likely pathogenic changes

The rest of the families comprising the group with novel very likely pathogenic changes present only single mutations. This is the case in families RP358B, RP60, RP180M, RP33 and RP81, with p.H2454PfsX8, p.Q27RfsX16, p.G288DfsX21, p.W1484X andp.N745S mutations, respectively (Table 2, Fig. 4).

### Families with novel possible pathogenic changes

The group of families with possibly pathogenic variations is composed of 2 families bearing missense variations which did not appear in 200 control individuals and affect important domains of the protein (VRP8 and RP383, Table 2 and Fig. 4), and 3 additional families with variations in the 5' UTR segment of *EYS* (RP228B, RP107B and RP509M, Table 2 and Fig. 4). Of these, transversion c.-462G>C deserves special interest as it has been identified in a homozygous state in the proband of the consanguineous family RP228B. The hypothesized pathogenic potential for this change would ensue from its position in regulatory sequences important for protein translation [Scheper et al., 2007]. Moreover, family segregation is compatible with disease.

Considering only the most likely pathogenic variations (truncating, stop, and frameshift), a prevalence estimate of 9.6% of distinct *EYS* variants in the Spanish arRP population can be drawn. Additionally, if the very likely pathogenic changes are included in the prevalence estimation, the figure could rise up to 15.9%. In an additional study performed in a separate population from United Kingdom, we have recently published eleven other novel mutations within *EYS* with probable allele frequency of 11 % [Abd El-Aziz et al., 2010].

Furthermore, it is worth mentioning that many of the domains that feature EYS protein are targeted by pathogenic variations. Yet, different pathogenic mechanisms are postulated depending on the nature of the variation. Probably, in the case of variations generating a premature stop codon, most of these altered mRNA transcripts will be lost through Nonsense Mediated Decay (NMD) [Frischmeyer et al., 2002].

With regard to the coding mutations, there is not a clear clustering of both the previously reported mutations and those identified in this study to indicate a common target in the primary sequence of the protein. Although the distribution of these mutations implicated in arRP reveals 15 affected exons, a trend for alterations in residues of the C-terminal region containing alternating EGF-Laminin G domains of the protein is observed. Particularly, 4 out of the 6 missense variations are found in the Laminin G and EGF domains of the second half of the protein (Fig. 3). This is consistent with the hypothesis presented by other groups that the C-terminal region would be crucial for the function of the protein [Collin et al., 2008], and with the homology results presented in the current study, which outline the C-terminal region as one of the highly conserved intervals. Accordingly, the homology analysis presented here also reveals the high degree of evolutionary conservation of all the domains presenting altered residues by the mutations identified in arRP patients (Supp. Figure S1).

Interestingly, mutations p.I2239SfsX17, p.Y3135X and p.W2640X had been previously reported as disease causing in 2 Dutch and 1 Spanish family respectively [Abd El-Aziz et al., 2008; Collin et al., 2008]. We have performed an extensive haplotype analysis and based on all available marker data we get different genotype information associated with the mutant alleles. Therefore, it is very likely that they are recurring mutations. Identifying recurrent mutations in Caucasian and especially specific populations such as the Spanish one provides an essential source for the molecular and clinical diagnosis of such a heterogeneous disease. Furthermore, this fact reinforces our hypothesis that *EYS* is the first prevalent gene in arRP [Abd El-Aziz et al., 2008].

The identification of 6 missense variations within the disease related changes in *EYS* differs from the mainly deletion/truncation mutations reported in previous studies ([Abd El-Aziz et al., 2008; Collin et al., 2008]. Nonetheless, the identification of missense mutations in arRP patients have already been reported [Sun et al., 1997; Molday et al., 2000], suggesting a refinement of the model based on the observation that some missense alleles might behave as true null allele at the functional level and may be responsible for severe impairment of protein function. Furthermore, majority of missense mutations reported here are located in functional conserved EYS domains and are more prevalent in the domains of the second half of the protein, thereby indicating a pathologic role for such variants (Fig. 3, Supp. Figure S1).

It is noteworthy that a significant proportion of families in our study with an *EYS* mutation had only one identified mutation. Interestingly, structural variations have been found in 2 out of the 6 assessed patients. Besides, in the previous paper we had identified 2 large deletions which are not detectable by direct genomic sequencing [Abd El-Aziz et al., 2008] and this may explain why in a proportion of patients the second mutation remained unidentified as reported in the current work. Additional experiments consisting of Copy Number Variations (CNVs) or MLPA (Multiplex Ligation-dependent Probe Amplification) analysis would be useful to rule out long heterozygous deletions.

The identification of distinct mutations in *EYS* reveals a probable mutation frequency of 15.9% in the Spanish arRP population. Along with the detection of three recurrent mutations in Caucasian population, our hypothesis of *EYS* being the first prevalent gene in arRP has been reinforced in the present study.

## Supplementary material

**Supp. Figure S1.** *EYS* homologues protein alignment and domain distribution with identified variations. 5′ UTR and splice site variations are not included in this figure.
