# Supplementary figures and images for: Mutation Spectrum of EYS in Spanish Patients with Autosomal Recessive Retinitis Pigmentosa
Source: Hum Mutat. 2010 Nov;31(11):E1772–800. doi: 10.1002/humu.21334 (PMC3045506; doi:10.1002/humu.21334)

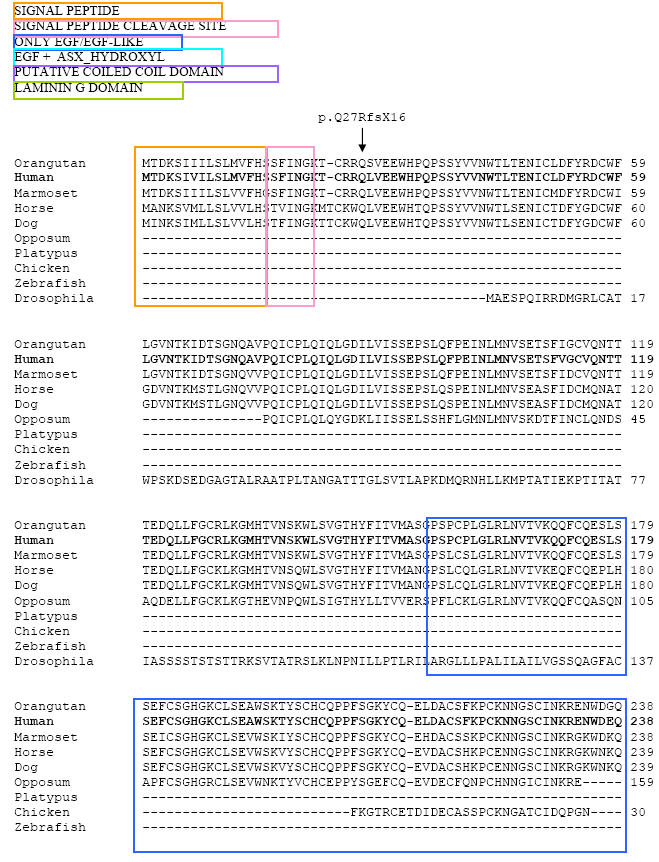

Supplement: Supplementary file 1 [file humu0031-E1772-SD1.gif]

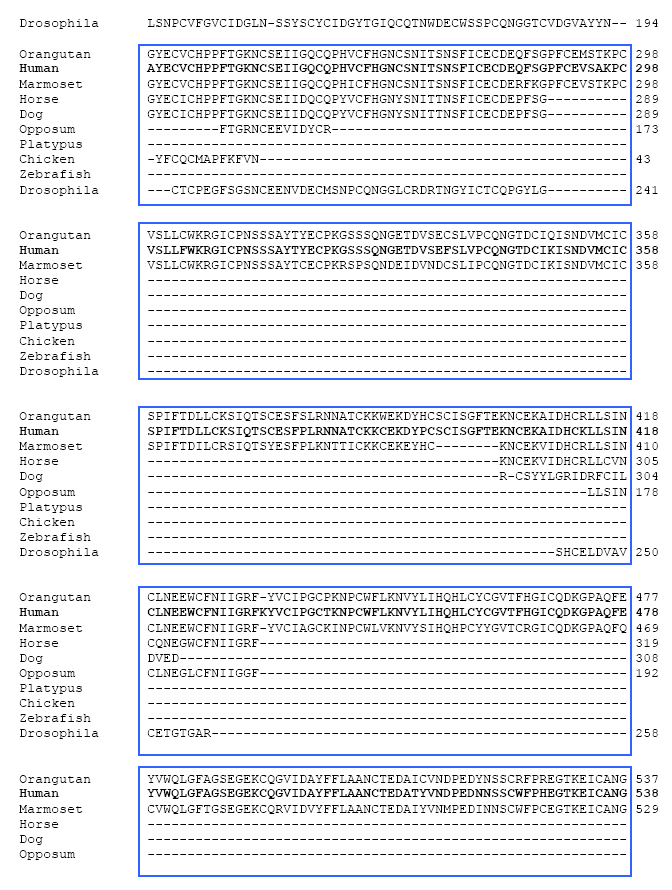

Supplement: Supplementary file 2 [file humu0031-E1772-SD2.gif]

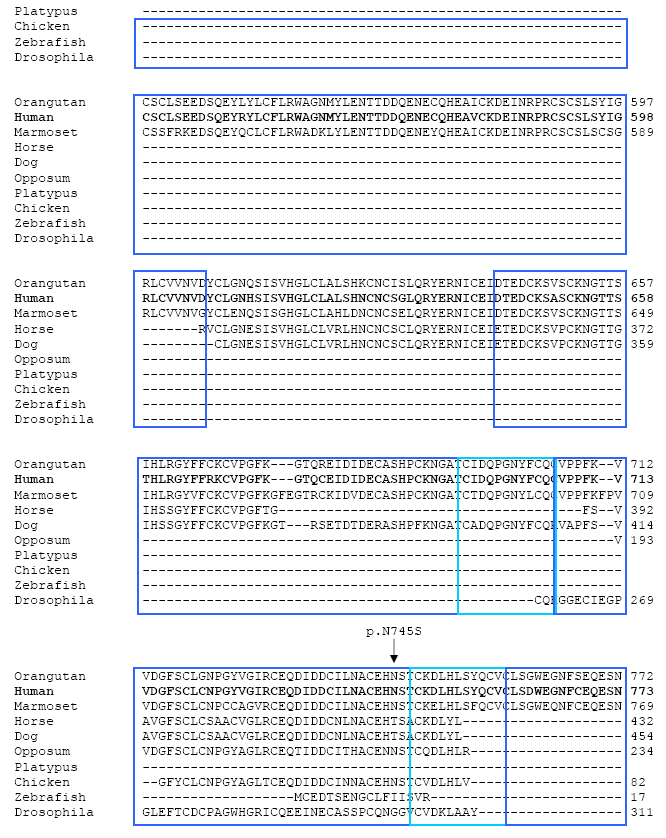

Supplement: Supplementary file 3 [file humu0031-E1772-SD3.gif]

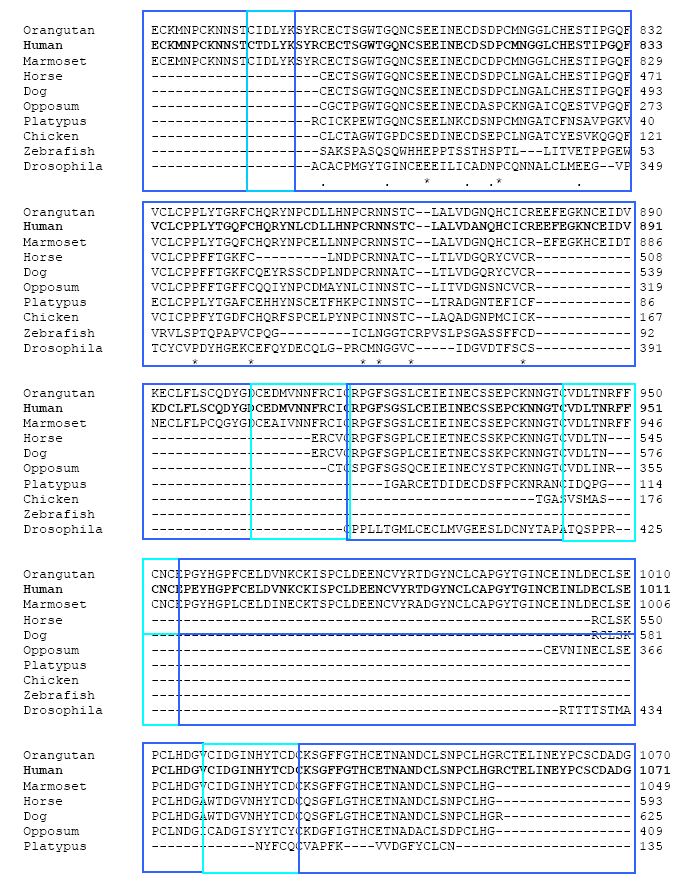

Supplement: Supplementary file 4 [file humu0031-E1772-SD4.gif]

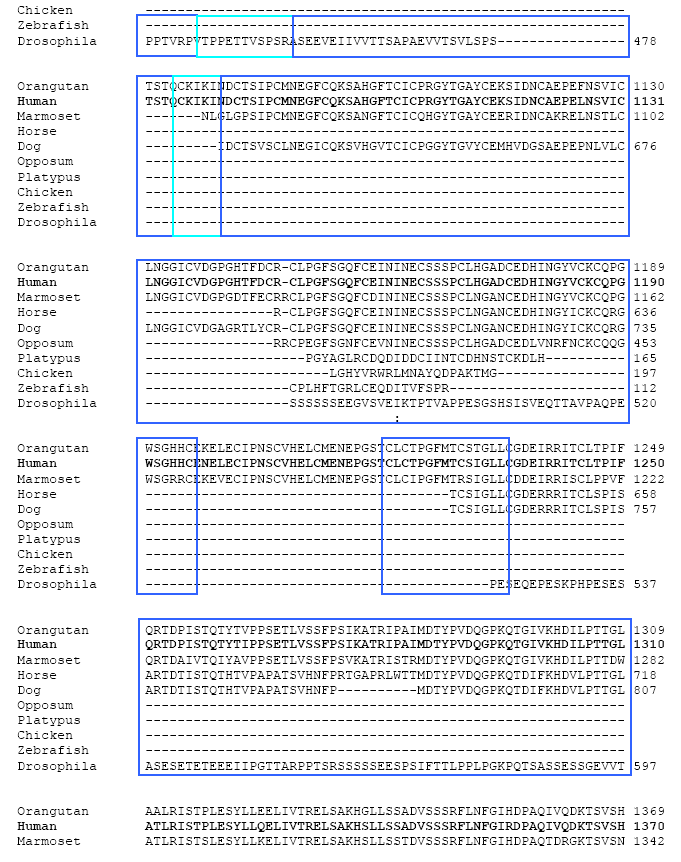

Supplement: Supplementary file 5 [file humu0031-E1772-SD5.gif]

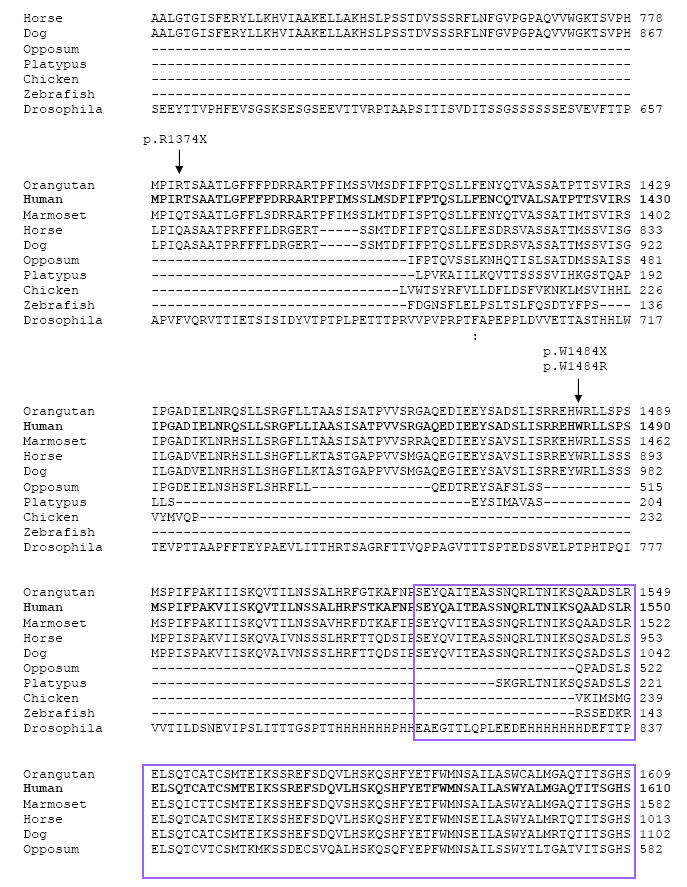

Supplement: Supplementary file 6 [file humu0031-E1772-SD6.gif]

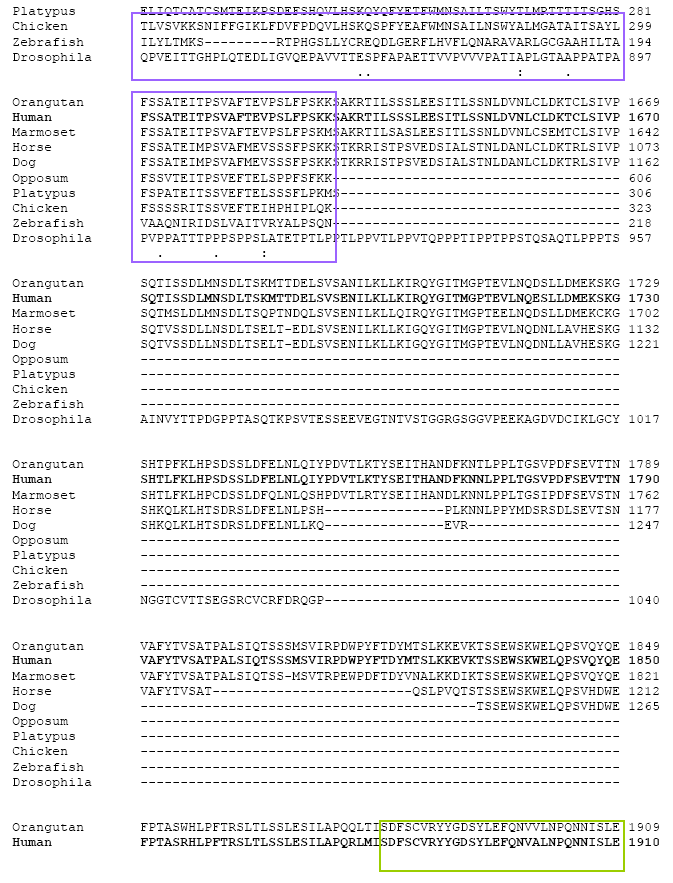

Supplement: Supplementary file 7 [file humu0031-E1772-SD7.gif]

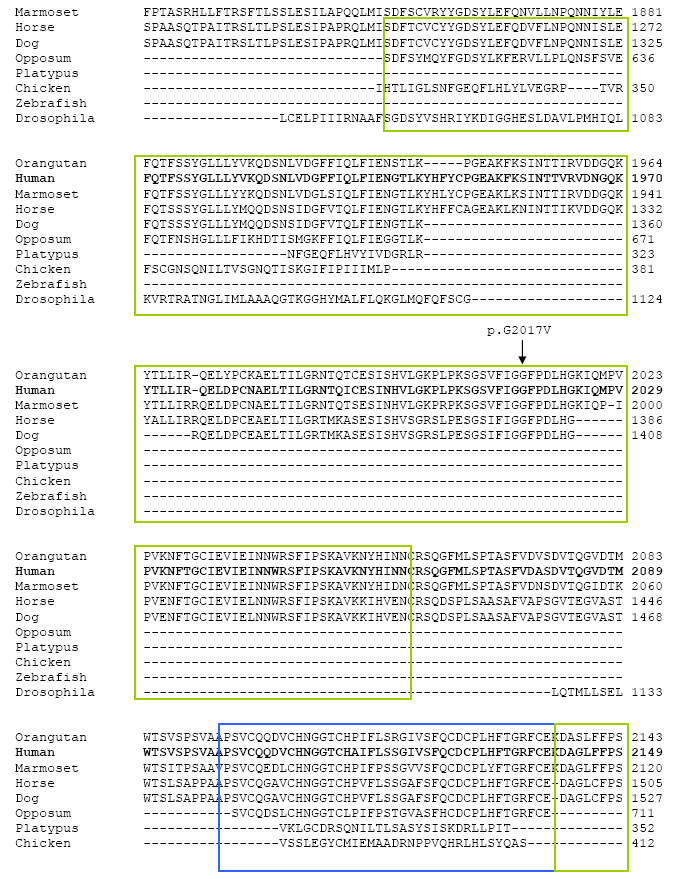

Supplement: Supplementary file 8 [file humu0031-E1772-SD8.gif]

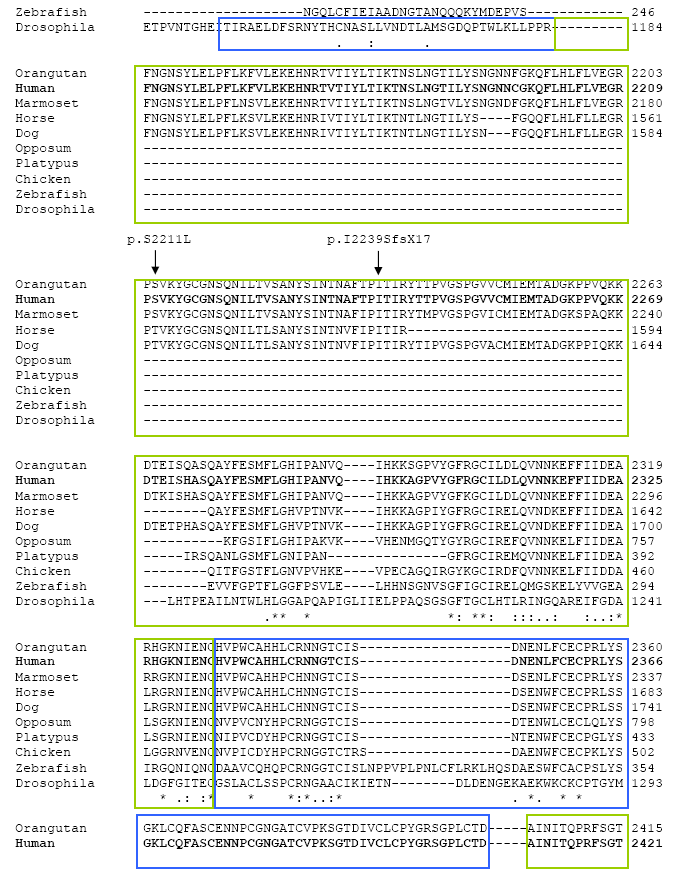

Supplement: Supplementary file 9 [file humu0031-E1772-SD9.gif]

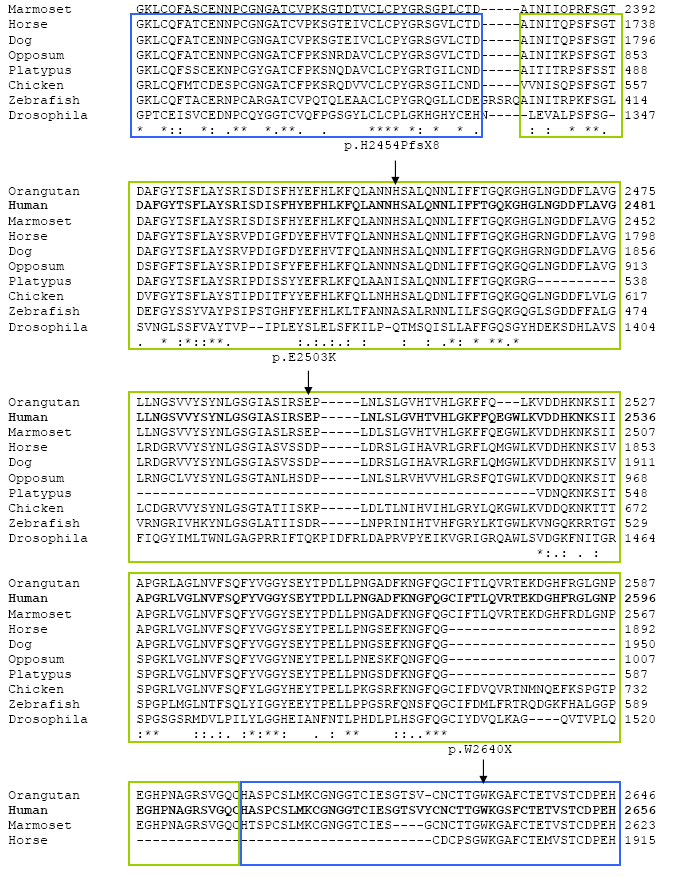

Supplement: Supplementary file 10 [file humu0031-E1772-SD10.gif]

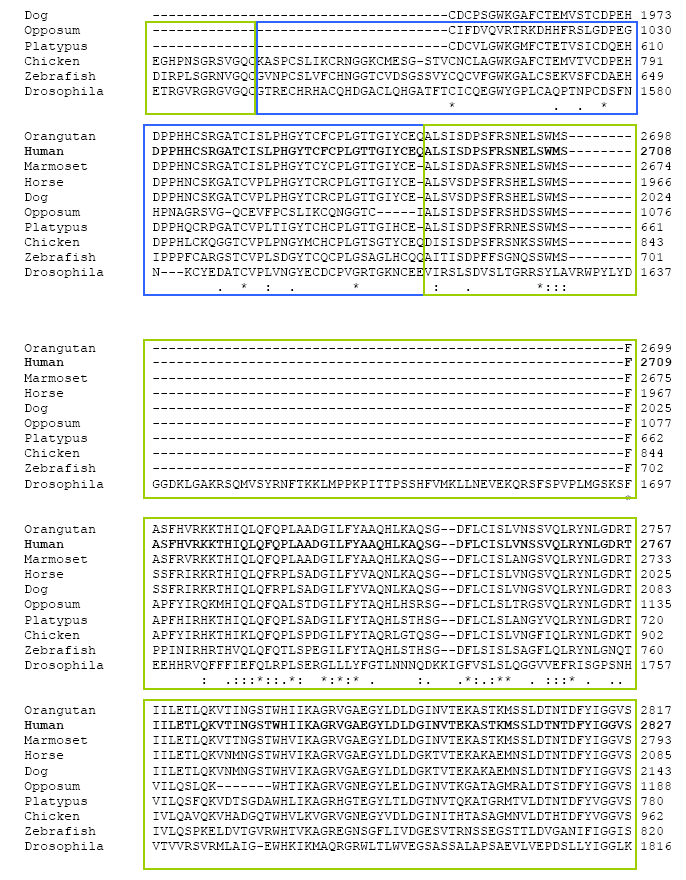

Supplement: Supplementary file 11 [file humu0031-E1772-SD11.gif]

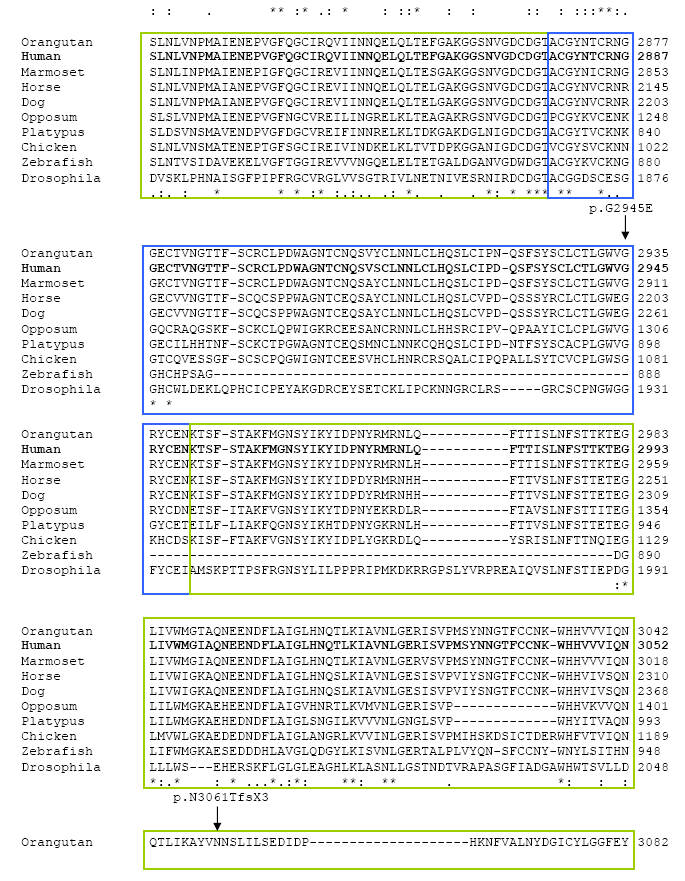

Supplement: Supplementary file 12 [file humu0031-E1772-SD12.gif]

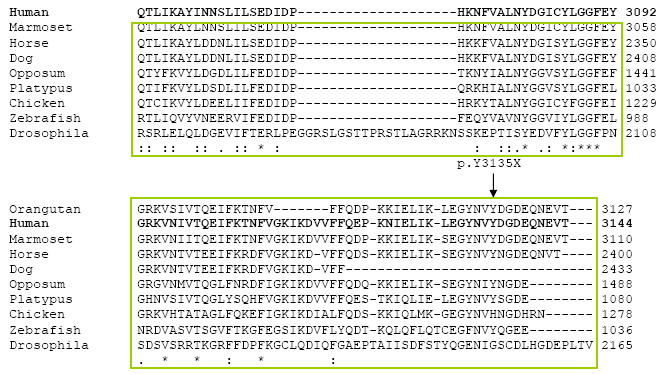

Supplement: Supplementary file 13 [file humu0031-E1772-SD13.gif]
